# Aberrant Autophagy Impacts Growth and Multicellular Development in a *Dictyostelium* Knockout Model of CLN5 Disease

**DOI:** 10.3389/fcell.2021.657406

**Published:** 2021-07-05

**Authors:** Meagan D. McLaren, Sabateeshan Mathavarajah, William D. Kim, Shyong Q. Yap, Robert J. Huber

**Affiliations:** ^1^Environmental and Life Sciences Graduate Program, Trent University, Peterborough, ON, Canada; ^2^Department of Biology, Trent University, Peterborough, ON, Canada

**Keywords:** autophagy, Batten disease, CLN5, development, *Dictyostelium discoideum*, glycoside hydrolase, growth, neuronal ceroid lipofuscinosis

## Abstract

Mutations in *CLN5* cause a subtype of neuronal ceroid lipofuscinosis (NCL) called CLN5 disease. While the precise role of CLN5 in NCL pathogenesis is not known, recent work revealed that the protein has glycoside hydrolase activity. Previous work on the *Dictyostelium discoideum* homolog of human CLN5, Cln5, revealed its secretion during the early stages of development and its role in regulating cell adhesion and cAMP-mediated chemotaxis. Here, we used *Dictyostelium* to examine the effect of *cln5*-deficiency on various growth and developmental processes during the life cycle. During growth, *cln5*^–^ cells displayed reduced cell proliferation, cytokinesis, viability, and folic acid-mediated chemotaxis. In addition, the growth of *cln5*^–^ cells was severely impaired in nutrient-limiting media. Based on these findings, we assessed autophagic flux in growth-phase cells and observed that loss of *cln5* increased the number of autophagosomes suggesting that the basal level of autophagy was increased in *cln5*^–^ cells. Similarly, loss of *cln5* increased the amounts of ubiquitin-positive proteins. During the early stages of multicellular development, the aggregation of *cln5*^–^ cells was delayed and loss of the autophagy genes, *atg1* and *atg9*, reduced the extracellular amount of Cln5. We also observed an increased amount of intracellular Cln5 in cells lacking the *Dictyostelium* homolog of the human glycoside hydrolase, hexosaminidase A (HEXA), further supporting the glycoside hydrolase activity of Cln5. This observation was also supported by our finding that *CLN5* and *HEXA* expression are highly correlated in human tissues. Following mound formation, *cln5*^–^ development was precocious and loss of *cln5* affected spore morphology, germination, and viability. When *cln5*^–^ cells were developed in the presence of the autophagy inhibitor ammonium chloride, the formation of multicellular structures was impaired, and the size of *cln5*^–^ slugs was reduced relative to WT slugs. These results, coupled with the aberrant autophagic flux observed in *cln5*^–^ cells during growth, support a role for Cln5 in autophagy during the *Dictyostelium* life cycle. In total, this study highlights the multifaceted role of Cln5 in *Dictyostelium* and provides insight into the pathological mechanisms that may underlie CLN5 disease.

## Introduction

The neuronal ceroid lipofuscinoses (NCLs), commonly known as Batten disease, comprise a family of rare neurodegenerative disorders that affect all ages and ethnicities (Dolisca et al., 2013). Batten disease is defined by two characteristics; (1) the accumulation of autofluorescent storage deposits in virtually every cell type and (2) the progressive loss of neurons in the cerebral cortex, cerebellum, and retina (Radke et al., 2015). The combination of storage body accumulation and neurodegeneration causes vision loss, seizures, loss of cognitive and motor function, and premature death (Radke et al., 2015). The cellular and molecular mechanisms underlying the NCLs are still unknown, largely because the normal functions of the 13 proteins associated with the disease (CLN1-8, CLN10-14) are not fully understood (Cárcel-Trullols et al., 2015). As a result, there is no curative therapy for any of the Batten disease subtypes. Mutations in ceroid neuronal lipofuscinosis protein 5 (*CLN5*) cause a late-infantile onset form of NCL known as CLN5 disease. Juvenile and adult cases have also been reported (Cannelli et al., 2007; Xin et al., 2010). CLN5 is a soluble lysosomal glycoside hydrolase that is secreted (Isosomppi et al., 2002; Moharir et al., 2013; Hughes et al., 2014; Huber and Mathavarajah, 2018a, b). Research has speculated that CLN5 may function in sphingolipid transport, lipid metabolism, lysosome receptor sorting, adhesion, endosomal retromer recruitment, mitophagy, and autophagy (Haddad et al., 2012; Mamo et al., 2012; Schmiedt et al., 2012; Leinonen et al., 2017; Huber and Mathavarajah, 2018a, b; Adams et al., 2019; Doccini et al., 2020).

The localizations and functions of NCL proteins have been studied in a variety of mammalian and non-mammalian systems (Huber et al., 2020a). One of these model systems is *Dictyostelium discoideum*, which is a soil-living eukaryotic microbe that is an exceptional organism for studying a variety of fundamental cellular and developmental processes (Huber, 2016, 2020; Mathavarajah et al., 2017; McLaren et al., 2019). The *Dictyostelium* genome encodes homologs of 11 of the 13 human NCL proteins, including a homolog of the TBC domain-containing protein kinase-like (TBCK) protein, which has been linked to a new possible subtype of NCL (CLN15 disease) (Huber, 2016; Beck-Wödl et al., 2018). In addition, *Dictyostelium* is the only lower eukaryotic model system that contains homologs of human tripeptidyl peptidase (TPP1, also known as CLN2) and CLN5 (Huber, 2016). Homologs of human TPP1/CLN2, CLN3, CLN5, and major facilitator superfamily domain-containing protein 8 (MFSD8, also known as CLN7) have been previously studied in *Dictyostelium*, which has contributed to our understanding of the localizations and functions of these proteins in human cells (Huber et al., 2014, 2017, 2020b; Phillips and Gomer, 2015; Huber, 2017, 2020; Stumpf et al., 2017; Huber and Mathavarajah, 2018a, b, 2019; Mathavarajah et al., 2018; Smith et al., 2019). Like human CLN5, *Dictyostelium*, Cln5 is secreted and has glycoside hydrolase activity (Huber and Mathavarajah, 2018a, b). Previous work revealed the Cln5 interactome in *Dictyostelium* and showed that Cln5 plays a role in regulating adhesion and cAMP-mediated chemotaxis (Huber and Mathavarajah, 2018a, b).

In nutrient-rich conditions, *Dictyostelium* amoebae grow as single cells, multiply by mitosis, and obtain nutrients by endocytosis (Mathavarajah et al., 2017). In nature (and in the laboratory), *Dictyostelium* cells find their food source by chemotactically responding to folic acid that is secreted by bacteria. Removal of nutrients places cells in a starved state and prompts a 24-h multicellular developmental program that relies on autophagy to generate energy and facilitate cell death during terminal differentiation. Cells first undergo chemotactic aggregation toward cyclic adenosine monophosphate (cAMP) to form multicellular mounds. Mounds develop into finger-like projections that rise above the surface only to fall back to the surface as motile pseudoplasmodia, or slugs. Cell populations within each slug terminally differentiate to form a fruiting body composed of a stalk of vacuolized cells that supports a mass of spores. When nutrients become available, the spores germinate to restart the life cycle. Owing to its dynamic life cycle, research in *Dictyostelium* has contributed to our understanding of conserved eukaryotic processes including chemotaxis, morphogenesis, differentiation, and autophagy, among others (Jeon et al., 2009; Uchikawa et al., 2011; Loomis, 2014; Mesquita et al., 2017).

Under basal conditions, and when nutrients are limited, cells undergo autophagy to break down and recycle their own reserves as a source of energy (e.g., organelles) (Mizushima, 2009). Autophagy has been thoroughly studied in *Dictyostelium* and many assays have been developed to study the process in detail (Domínguez-Martín et al., 2017). When autophagy is activated, an elongating double membrane known as the phagophore engulfs intracellular cargo (protein aggregates, misfolded proteins, and defective organelles) and then closes to become an autophagosome. The outer membrane of the autophagosome then fuses with the lysosome to form an autolysosome, leading to autophagic degradation of the autophagosomal inner membrane and cargo by lysosomal hydrolases. The rate of turnover of autophagosomes and the efficiency of autophagic degradation is known as autophagic flux (Mizushima et al., 2010). Autophagy occurs throughout the *Dictyostelium* life cycle. As a result, *Dictyostelium* serves as a powerful model for exploring autophagic processes at the single cell and multicellular levels.

In this study, we examined the role of Cln5 in regulating growth and multicellular development in *Dictyostelium*. During growth, we assessed the effect of *cln5*-deficiency on cell proliferation, cytokinesis, pinocytosis, viability, and folic acid-mediated chemotaxis. We then linked these growth-phase defects to aberrant autophagy in *cln5*^–^ cells. We also examined the development of *cln5*^–^ cells to determine the effects of *cln5*-deficiency on aggregation and the timing of development, which we also linked to aberrant autophagy in *cln5*^–^ cells. Finally, we provide additional evidence supporting the glycoside hydrolase activity of Cln5 in *Dictyostelium*. In total, our findings are consistent with a role for Cln5 in regulating autophagy during *Dictyostelium* growth and multicellular development.

## Materials and Methods

### Cell Lines, Media, and Buffers

AX3 cells, hereafter referred to as wild type (WT), were the parental line for *cln5*^–^ cells (Huber and Mathavarajah, 2018b). The *cln5*^–^ cell line expressing Cln5 in pDM323 (*cln5*^–^ + Cln5-GFP) was generated and validated in a previous study (Huber and Mathavarajah, 2018b). WT and *cln5*^–^ cells expressing either pA15/GFP-Atg8a or pA15/RFP-GFP-Atg8a (constructs purchased from the Dicty Stock Center) were generated using a transformation protocol described elsewhere (Otto et al., 2004; Fey et al., 2007, 2013; Calvo-Garrido et al., 2011). *atg1*^–^ (DBS0236346), *atg9*^–^ (DBS0305977), and *nagA*^–^ (DBS0235654) cells were purchased from the Dicty Stock Center (Dimond et al., 1973; Otto et al., 2004; Tung et al., 2010; Fey et al., 2013). The parental lines for these cells were also purchased from the Dicty Stock Center and are referred to as WT for the experiments where they were used (KAX3 for *atg1*^–^, DBS0236345; AX2 for *atg9*^–^, DBS0235525; AX3 for *nagA*^–^, DBS0235542). Cells were maintained on SM/2 agar with *Klebsiella aerogenes* at 22°C (Fey et al., 2007). Cells used for experiments were grown axenically in nutrient-rich medium (HL5) at 22°C with shaking at 150 rpm. HL5, minimal medium (FM), FM lacking amino acids (FM-aa), and low-fluorescence HL5 were purchased from Formedium (Hunstanton, Norfolk, United Kingdom). Cultures were supplemented with ampicillin (100 μg/ml) and streptomycin sulfate (300 μg/ml) to prevent bacterial growth (Bioshop Canada Incorporated, Burlington, ON, Canada). *cln5*^–^ cells required the addition of blasticidin S hydrochloride (10 μg/ml) for selection and cell lines carrying extrachromosomal vectors (e.g., pA15/GFP-Atg8a) required the addition of Geneticin (G418) (10 μg/ml) (Bioshop Canada Incorporated, Burlington, ON, Canada) (Levi et al., 2000; Veltman et al., 2009; Faix et al., 2013). KK2 buffer was composed of 2.2 g/l KH_2_PO_4_ and 0.7 g/l K_2_HPO_4_, pH 6.5.

### Antibodies and Chemicals

Mouse monoclonal anti-GFP and mouse monoclonal anti-beta-actin were purchased from Santa Cruz Biotechnology Incorporated (Dallas, TX, United States). Mouse monoclonal P4D1 anti-ubiquitin was purchased from New England Biolabs Canada (Whitby, ON, Canada). Mouse monoclonal anti-alpha-actinin (47-62-17) and mouse monoclonal anti-alpha-tubulin (12G10) were purchased from the Developmental Studies Hybridoma Bank (University of Iowa, Iowa City, IA, United States). The generation and validation of anti-Cln5 and anti-CtsD were described elsewhere (Huber and Mathavarajah, 2018b; Huber et al., 2020b). Anti-countin (CtnA) was provided as a gift by Dr. Richard Gomer (Brock and Gomer, 1999). A polyclonal antibody was raised in rabbits against a synthetic peptide corresponding to the epitope ^187^KDETFPKNMTVTQD^200^ in the C-terminal region of *Dictyostelium* calcium-dependent cell adhesion molecule A (CadA) (Genscript, Piscataway, NJ, United States). The antibody was affinity purified and validated using a peptide competition assay (Huber et al., 2020b; Supplementary Figure 1). Briefly, a 1:67 dilution of anti-CadA was prepared in 1 ml of western blotting washing buffer (TBST) ± the peptide epitope (500 μg). The solutions were then incubated overnight at 4°C with head over tail rotation, after which time the tubes were spun at 14000 rpm for 5 min. The supernatants were then added to 2% milk/TBST solution for western blotting. Goat anti-rabbit and horse anti-mouse IgG linked to HRP were purchased from New England Biolabs Canada (Whitby, ON, Canada). Alexa-488 Fluor-conjugated secondary antibodies were purchased from Fisher Scientific Company (Ottawa, ON, Canada). FITC-dextran and folic acid were purchased from Sigma-Aldrich Canada (Oakville, ON, Canada). Ammonium chloride was purchased from Bioshop Canada Incorporated (Burlington, ON, Canada). Prolong Gold Anti-Fade Reagent with DAPI was purchased from Fisher Scientific Company (Ottawa, ON, Canada).

### Cell Proliferation, Cytokinesis, Pinocytosis, and Viability Assays

Growth assays were performed using a method described elsewhere (Huber et al., 2014). Briefly, cells in the mid-log phase of growth (1–5 × 106 cells/ml) were diluted to 1 × 105 cells/ml in fresh HL5, FM, or FM-aa at 22°C with shaking at 150 rpm. Every 24 h for 4–5 days, the densities of cell suspensions were measured (in triplicate aliquots) using a hemocytometer. For the cytokinesis assay, cells (2 × 10^5^ total) in the mid-log phase of growth (1–5 × 10^6^ cells/ml) were deposited onto coverslips placed inside separate wells of a 12-well dish. Coverslips were then submerged in low-fluorescence HL5 (to reduce background fluorescence) and incubated overnight at 22°C. Cells were fixed in −80°C methanol for 45 min (Hagedorn et al., 2006) and mounted on slides with Prolong Gold Anti-Fade Reagent with DAPI. Cells were imaged using a Nikon Ts2R-FL inverted microscope equipped with a Nikon Digital Sight Qi2 monochrome camera (Nikon Canada Incorporated Instruments Division, Mississauga, ON, Canada). The number of nuclei within each cell was scored. For each experiment, 10 random images were obtained from each coverslip and each image contained at least 10 cells. The pinocytosis assay was conducted using a method described elsewhere (Rivero and Maniak, 2006; Huber et al., 2014). Briefly, cells in the mid-log phase of growth (1–5 × 10^6^ cells/ml) were placed in fresh HL5 at a density of 5.0 × 10^6^ cells/ml. After 15 min, 100 μl of a 20 mg/ml FITC-dextran (70,000 M_r_) stock solution was added to the suspension generating a final concentration of 400 μg/ml, respectively. Every 15 min over a 90-min period, 500 μl of cells were collected, washed twice with 500 μl of ice-cold Sorenson’s buffer (2 mM Na_2_HPO_4_, 14.6 mM KH_2_PO_4_, pH 6.0) and lysed with 1 ml of pinocytosis lysis buffer (50 mM Na_2_HPO_4_, pH 9.3, 0.2% Triton-X). Lysates (100 μl) were added to separate wells of a black 96-well plate and fluorescence was measured using a BioTek Synergy HTX plate reader (excitation 470 nm, emission 515 nm) (BioTek Instruments Incorporated, Winooski, VT, United States). To assess cell viability, cells were grown in FM and FM-aa according to the growth assay protocol described above. The CellTiter-Glo Luminescent Cell Viability Assay was then used according to the manufacturer’s instructions (Promega Corporation, Madison, WI, United States). Aliquots of 50 μl were taken from each culture at the indicated time points. These aliquots were then quickly added to separate wells of a black 96-well plate each containing 50 μl of CellTiter-Glo reagent. The solution was incubated at 22°C for 20 min after which time luminescence was measured using a BioTek Synergy HTX microplate reader.

### Radial Bioassay of Chemotaxis Toward Folic Acid

Chemotaxis toward folic acid was assessed using a radial bioassay (O’Day, 1979). Briefly, cells in the mid-log phase of growth (1–5 × 10^6^ cells/ml) were grown in HL5 overnight to confluency in Petri dishes. The following day, cells were harvested, washed two times with KK2 buffer, and plated (1 × 10^8^ cells/ml) in 0.6 μl volumes on 0.5% agar/KK2 buffer ± folic acid (50 μM). Cell spots were imaged at 0 and 5 h using a Nikon Ts2R-FL inverted microscope equipped with a Nikon Digital Sight Qi2 monochrome camera. Images were viewed using NIS Elements Basic Research and analyzed using Fiji/ImageJ (Schneider et al., 2012).

### Autophagic Flux Assay

Wild type and *cln5*^–^ cells expressing GFP-Atg8a in the mid-log phase of growth (1–5 × 10^5^ cells/ml) were grown in HL5 overnight to confluency in Petri dishes. The following day, cells were washed twice with KK2 buffer and then submerged in fresh HL5 ± ammonium chloride (150 mM) for 4 h. Treatment with ammonium chloride elevates the pH of the lysosome, thereby inhibiting autophagy (Calvo-Garrido et al., 2011). An additional pulse of ammonium chloride (150 mM) was added halfway through the treatment (after 2 h). After 4 h, cells were harvested, lysed with NP40 lysis buffer (150 mM NaCl, 50 mM Tris, 0.5% NP-40, pH 8.3) containing a protease cocktail inhibitor tablet (Fisher Scientific Company, Ottawa, ON, Canada), and analyzed for GFP-Atg8a cleavage by western blotting. WT and *cln5*^–^ cells expressing RFP-GFP-Atg8a in the mid-log phase of growth (1–5 × 10^5^ cells/mL) were deposited onto coverslips (5.0 × 10^5^ total) submerged in low fluorescence HL5 (in separate wells of a 12-well dish) and incubated overnight at 22°C. The following day, cells were incubated in fresh low-fluorescence HL5 ± ammonium chloride (150 mM) for 4 h. An additional pulse of ammonium chloride (150 mM) was added halfway through the treatment (after 2 h) (Domínguez-Martín et al., 2017). Cells were fixed in −80°C methanol for 45 min (Hagedorn et al., 2006) and mounted on slides with Prolong Gold Anti-Fade Reagent with DAPI so that they could be viewed by epifluorescence microscopy. Cells were imaged using a Leica DM6000B microscope equipped with a Leica DFC350FX digital camera (Leica Microsystems Canada Incorporated, Richmond Hill, ON, Canada). Images were viewed and merged using Fiji/ImageJ. To assess the effect of *cln5*-deficiency on the levels of ubiquitin-positive proteins, WT and *cln5*^–^ cells in the mid-log phase of growth (1–5 × 10^5^ cells/ml) were grown in HL5 overnight to confluency in Petri dishes. The following day, cells were washed twice with KK2 buffer and then submerged in fresh HL5 ± ammonium chloride (100 mM) for 2 h. Cells were then harvested, lysed with NP40 lysis buffer, and analyzed by western blotting to assess the levels of ubiquitin-positive proteins in WT and *cln5*^–^ cells.

### Aggregation Assay

Cells in the mid-log phase of growth (1−5 × 10^6^ cells/ml) were grown in HL5 overnight to confluency in Petri dishes. The following day, cells were washed twice with KK2 buffer and then submerged in fresh KK2 buffer. Starving cells were imaged using a Nikon Ts2R-FL inverted microscope equipped with a Nikon Digital Sight Qi2 monochrome camera. Cells were also harvested after 4 h of starvation, lysed with NP40 lysis buffer, and analyzed by western blotting to assess the effect of *cln5*-deficiency on the intracellular and extracellular amounts of CadA, CtnA, and CtsD, as well as the intracellular and extracellular amounts of Cln5 in *atg1*^–^ and *atg9*^–^ cells. In separate experiments, 5 × 10^6^ cells were deposited into 6 well dishes and allowed to adhere for 30 min. Cells were then washed twice with FM-aa and incubated in fresh FM-aa for 24 h. Mounds were imaged after 24 h using a Nikon Ts2R-FL inverted microscope equipped with a Nikon Digital Sight Qi2 monochrome camera.

### Multicellular Development Assay

Multicellular development was assessed on two substrates using previously described methods: (1) black, gridded, nitrocellulose filters (0.45 mm pore size) (Fisher Scientific Company, Ottawa, ON, Canada) and (2) 0.5% agar/distilled water ± ammonium chloride (60 mM) (Huber et al., 2014). For development on nitrocellulose filters, developmental chambers were prepared in 60 mm × 15 mm Petri dishes. In each chamber, a black gridded nitrocellulose filter was placed on top of four Whatman #3 cellulose filters (Fisher Scientific Company, Ottawa, ON, Canada) pre-soaked in KK2 buffer. For development on 0.5% agar/distilled water ± ammonium chloride (60 mM), 5 ml of agar was poured into 60 mm × 15 mm Petri dishes. For both substrates, confluent cells grown in HL5 overnight on Petri dishes were washed twice with KK2 buffer. Washed cells (3 × 10^7^ cells/ml) were deposited in 25 μl droplets (750,000 cells total) on nitrocellulose filters or 0.5% agar/distilled water ± ammonium chloride (60 mM). During multicellular development, cells were maintained in a dark humidity chamber at 22°C. Developmental structures were imaged using a Leica EZ4W stereomicroscope equipped with an internal 5MP CMOS camera (Leica Microsystems Canada Incorporated, Richmond Hill, ON, Canada). For development on nitrocellulose filters, the percentage of respective morphological structures in each cell droplet was quantified at specific developmental time points. Slug size on 0.5% agar/distilled water ± ammonium chloride (60 mM) was quantified using Fiji/ImageJ.

### Spore Morphology, Germination, and Viability

To assess spore morphology, spores were collected from fresh fruiting bodies (24–48 h after development) and suspended in KK2 buffer (1 × 10^6^ spores/ml). Suspensions were then deposited in 60 mm × 15 mm Petri dishes and imaged using a Nikon Ts2R-FL inverted microscope equipped with a Nikon Digital Sight Qi2 monochrome camera. The area and roundedness of spores were analyzed using Fiji/ImageJ. For roundedness, a value of 1.0 indicated a completely circular spore. Statistical significance was determined using the Kruskal–Wallis test followed by Dunn’s multiple comparisons test. To examine germination, spores were collected and deposited in Petri dishes containing HL5. Spores within a specific field-of-view were examined every 10 min using a Nikon Ts2R-FL inverted microscope equipped with a Nikon Digital Sight Qi2 monochrome camera. When spores appeared to swell, marking the beginning of germination, the time was noted (Cotter and Raper, 1966). Statistical significance was determined using one-way ANOVA followed by Bonferroni’s multiple comparisons test. Finally, spore viability was assessed using a previously described method (Myre et al., 2011). Briefly, 50 spores were collected and spread with a fixed amount of *K. aerogenes* on SM/2 agar. After 4 days, the number of plaques formed on the bacterial lawn were counted to assess how many of the initial 50 spores were viable (i.e., how many germinated). Statistical significance was determined using the Kruskal–Wallis test followed by Dunn’s multiple comparisons test.

### SDS-PAGE and Western Blotting

Proteins in whole cell lysates and samples of conditioned buffer were quantified using a Qubit 2.0 Fluorometer (Fisher Scientific Company, Ottawa, ON, Canada). SDS-PAGE and western blotting were performed using standard methods (2-h incubation at 22°C for primary and secondary antibodies in 5% milk/TBST). The following antibody dilutions were used; anti-GFP (1:1000); anti-beta-actin (1:1000); anti-ubiquitin (1:1000); anti-alpha-actinin (1:1000); anti-alpha-tubulin (1:1000); anti-Cln5 (1:500); anti-CtsD (1:500); anti-CtnA (1:1000); anti-CadA (1:1000); anti-rabbit and anti-mouse IgG linked to HRP (1:5000). Protein bands were imaged using the enhanced chemiluminescence clarity max substrate and the ChemiDoc Imaging System (Bio-Rad Laboratories Limited, Mississauga, ON, Canada). Protein bands were quantified using Fiji/ImageJ.

### Statistical Analyses

All statistical analyses were performed using GraphPad Prism 8 (GraphPad Software Incorporated, La Jolla, CA, United States). A *p*-value < 0.05 was considered significant for all analyses and n represents the number of biological replicates analyzed. Details on the specific statistical analyses that were performed are found in the individual figure captions.

## Results

### *cln5*-Deficiency Inhibits Cell Proliferation and Cytokinesis in Nutrient-Rich Media but Has No Effect on Pinocytosis

**FIGURE 1:**
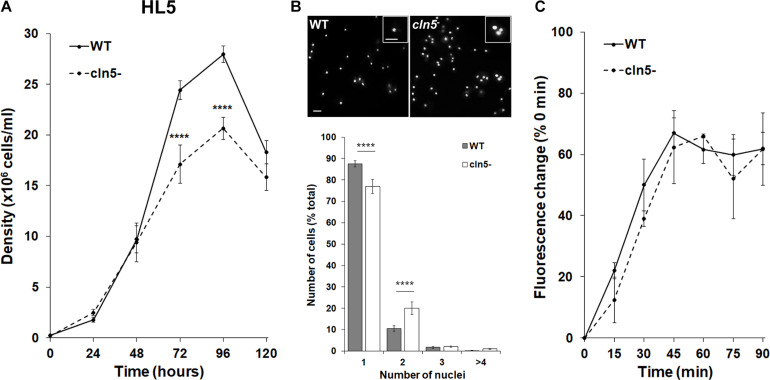
Effect of *cln5*-deficiency on cell proliferation, cytokinesis, and pinocytosis in HL5. **(A)** WT and *cln5*^−^ cells were placed in HL5 at 1.0 × 10^5^ cells/ml. Cell densities were measured every 24 h for 120 h. Data presented as mean density ± SEM (*n* = 11). Statistical significance was assessed using two-way ANOVA followed by Bonferroni’s multiple comparisons test. ****p-value < 0.0001 at the indicated time points vs. WT. **(B)** Growth-phase WT and *cln5*^−^ cells were fixed on coverslips and stained with DAPI to reveal nuclei. The percentage of cells in the population containing 1, 2, 3, or ≥4 nuclei was measured. Data presented as mean number of cells (% total) ± SEM (*n* = 6). Statistical significance was determined using the Student’s *t*-test. ****p-value < 0.0001 vs. WT. Scale bar = 10 μm. **(C)** WT and *cln5*^−^ cells (5.0 × 10^6^ cells/ml) were incubated in HL5 containing FITC-dextran (400 μg/ml). Starting at 0 min, and every 15 min thereafter, cells were collected, washed with ice-cold Sorenson’s buffer, and lysed. The fluorescence of the lysates was then measured. Data presented as mean fluorescence change (% 0 min) ± SEM (*n* = 3).

To examine the role of Cln5 during *Dictyostelium* growth, we assessed the effect of *cln5*-deficiency on cell proliferation in nutrient-rich media (HL5). *cln5*^–^ cell proliferation was significantly reduced after 72 and 96 h of growth, causing *cln5*^–^ cells to reach a lower maximum cell density (Figure 1A). During the assay, we observed that *cln5*^–^ cultures had comparatively more multinucleated cells compared to WT. To confirm this observation, *cln5*^–^ cells grown in suspension were fixed and stained with DAPI to reveal nuclei and assess the effect of *cln5*-deficiency on cytokinesis. The number of mono-nucleated, di-nucleated, tri-nucleated, and poly-nucleated (>3) cells were then scored. In *cln5*^–^ cell populations, 77 ± 3% of the cells had one nucleus compared to 88 ± 2% of WT cell populations (Figure 1B). Conversely, *cln5*^–^ cell populations had a greater number of di-nucleated cells compared to WT cell populations (20 ± 3% compared to 11 ± 1%, respectively) (Figure 1B). Next, we reasoned that the reduced proliferation of *cln5*^–^ cells could have potentially been due to a decreased rate of extracellular nutrient uptake known as pinocytosis. Therefore, we assessed the rate of pinocytosis of FITC-dextran by WT and *cln5*^–^ cells. In this assay, cells were incubated with FITC-dextran and intracellular FITC-dextran fluorescence was measured every 15 min over a 90-min period (Figure 1C). Two-way ANOVA did not reveal statistically significant differences between the cell lines indicating that the reduced proliferation of *cln5*^–^ cells was not due to aberrant pinocytosis. Combined, these data indicate that Cln5 plays a role in regulating cell proliferation and cytokinesis during *Dictyostelium* growth.

### Nutrient Limitation Severely Impacts the Proliferation and Viability of *cln5*^–^ Cells

**FIGURE 2:**
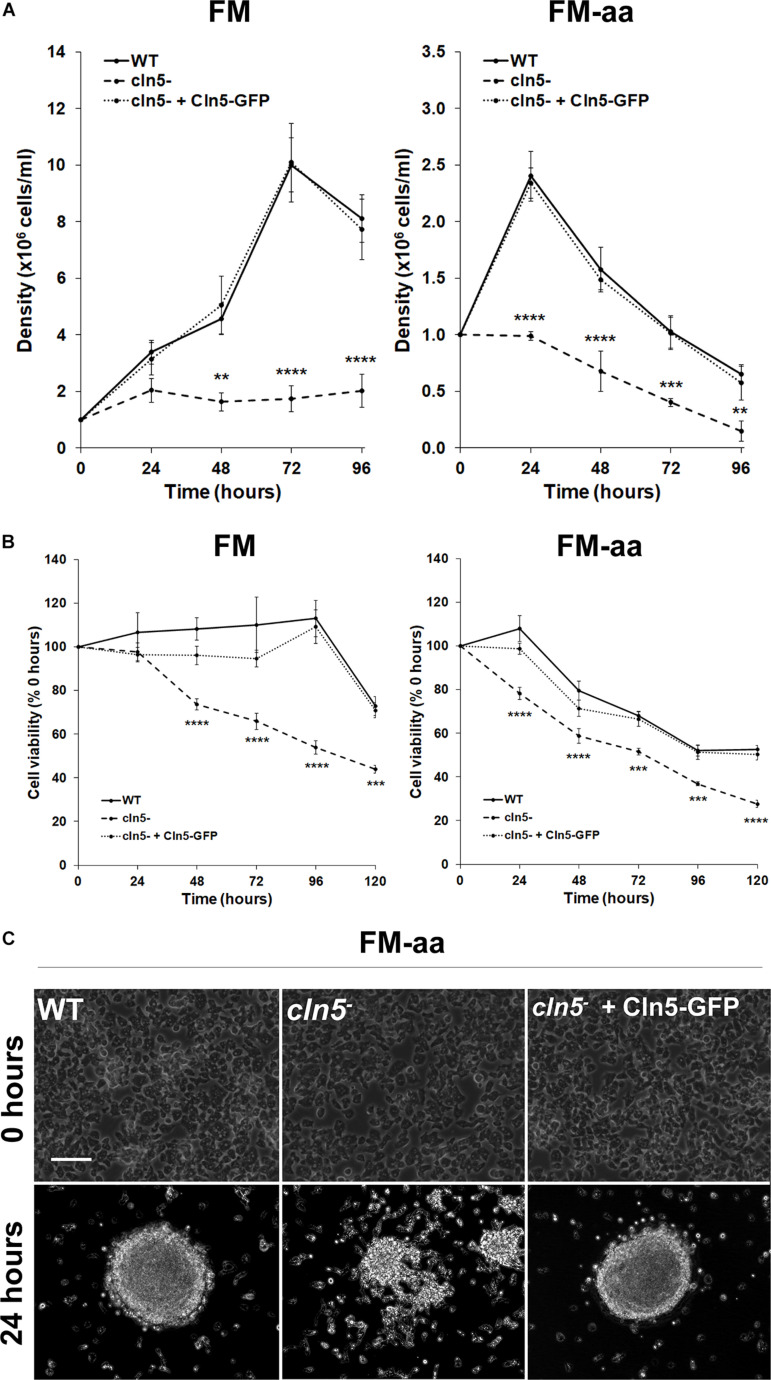
Effect of *cln5*-deficiency on cell proliferation and viability in FM and FM-aa. **(A)** WT, *cln5*^−^, and *cln5*^−^ + Cln5-GFP cells were placed in FM or FM-aa at a density of 1.0 × 10^5^ cells/ml. Cell densities were measured every 24 h for 96 h. Data presented as mean density ± SEM (*n* ≥ 4). Statistical significance was assessed using two-way ANOVA followed by Bonferroni’s multiple comparisons test. **p-value < 0.01, ***p-value < 0.001, and ****p-value < 0.0001 at the indicated time points vs. WT. **(B)** Cells were grown in FM and FM-aa as described in panel **(A)**. The CellTiter-Glo Luminescent Cell Viability Assay was used to quantify cell viability at the indicated time points. Statistical significance was determined using two-way ANOVA followed by Bonferroni’s multiple comparisons test. ***p-value < 0.001 and ****p-value < 0.0001 at the indicated time points vs. WT. **(C)** Cells (20 × 10^6^ total) were harvested from HL5 suspension and placed in a 100 × 15 mm Petri dish containing HL5. After 1 h, the HL5 was removed and replaced with FM-aa. Images were taken after 24 h and are representative of four independent experiments. Scale bar = 50 μm.

In nutrient-deprived states, *Dictyostelium* cells maintain their viability by generating nutrients through autophagy (Marin, 1976; Mesquita et al., 2017). Since the impaired proliferation of *cln5*^–^ cells in nutrient-rich media could not be explained by reduced pinocytosis, we were interested in assessing the effect of *cln5*-deficiency on cell proliferation in minimal media (FM) and minimal media lacking amino acids (FM-aa). FM is an alternate culturing media that has the minimal amounts of resources required for *Dictyostelium* growth (Franke and Kessin, 1977). FM-aa places cells in an environment where their survival is dependent on autophagy. WT and *cln5*^–^ cell densities were significantly reduced in both FM and FM-aa compared to their densities in HL5 (Figures 1A, 2A). In FM, *cln5*^–^ cell densities were significantly reduced after 48, 72, and 96 h compared to WT (Figure 2A). In contrast, the growth of WT cultures increased until 72 h, after which time they began to decrease (Figure 2A). In FM-aa, *cln5*^–^ cell densities were significantly reduced over the entire 96-h experimental period compared to WT cultures. The aberrant proliferation of *cln5*^–^ cells in both FM and FM-aa was restored by introducing Cln5-GFP into *cln5*^–^ cells (Figure 2A). Loss of *cln5* also significantly reduced the viability of cells cultured in FM and FM-aa, as determined by a commercially available kit that measures relative ATP levels (i.e., indicator of cell viability) (Figure 2B). Finally, we allowed WT, *cln5*^–^, and *cln5*^–^ + Cln5-GFP cells to adhere to the bottom of Petri dishes while submerged in FM-aa. After 24 h, WT and *cln5*^–^ + Cln5-GFP cells formed large circular mounds composed of tightly adhered cells (Figure 2C). In contrast, *cln5*^–^ cells were more dispersed, which was consistent with a previous study that reported an effect of *cln5*-deficiency on cell adhesion (Figure 2C; Huber and Mathavarajah, 2018b). Together, these findings indicated that Cln5 may play a role in autophagy during *Dictyostelium* growth.

### *cln5*-Deficiency Reduces Folic Acid-Mediated Chemotaxis

**FIGURE 3:**
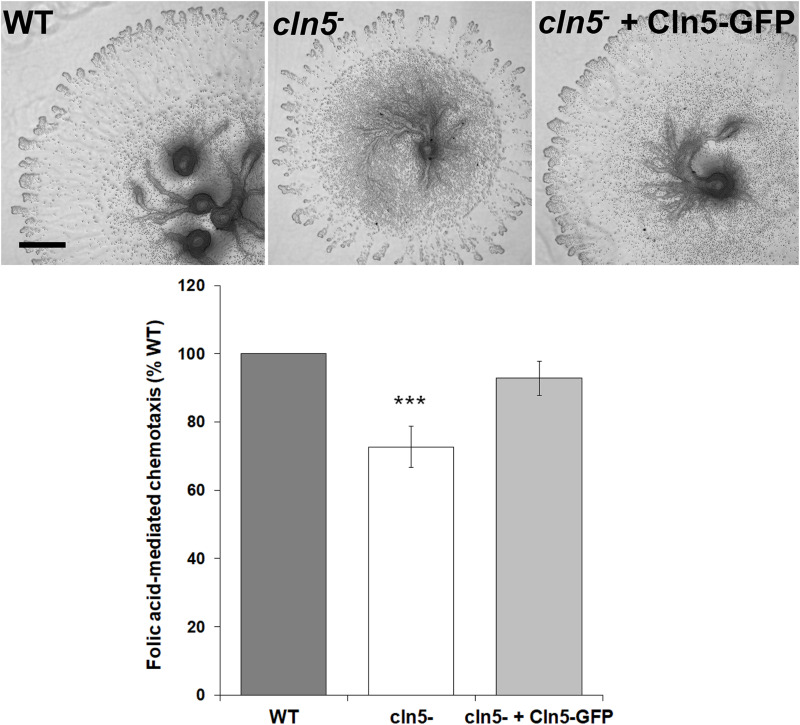
Effect of *cln5*-deficiency on folic acid-mediated chemotaxis. WT, *cln5*^−^, and *cln5*^–^ + Cln5-GFP cells were deposited onto 0.5% agar/KK2 buffer + folic acid (50 μM). Images were taken once cells were deposited and after 5 h. Scale bar = 500 μm. The amount of migration of each cell line was quantified and the raw values were then expressed as a percentage of the migration of WT cells. Data presented as mean folic acid-mediated chemotaxis (% WT) ± SEM (*n* ≥ 5). Statistical significance was assessed using the one sample *t*-test. ***p < 0.001 vs. WT.

Based on the previous results, we examined the effect of *cln5*-deficiency on folic acid-mediated chemotaxis since work in human cells lines (HEK-293 and U87 glioblastoma cells) supports a link between chemotaxis and autophagy, specifically autophagosome biogenesis (Coly et al., 2016). In addition, previous work showed that loss of *cln5* reduces cAMP-mediated chemotaxis during the early stages of *Dictyostelium* development (Huber and Mathavarajah, 2018b). For this assay, a droplet of cells was deposited on agar containing folic acid. After 5 h, the population of cells migrated outward forming a halo around the original deposition spot (Figure 3). The spot diameters were then quantified to determine the mean distance migrated by the cell population. Loss of *cln5* reduced folic acid-mediated chemotaxis by 27 ± 6% and introduction of Cln5-GFP into *cln5*^–^ cells restored the reduced chemotaxis (Figure 3). Together, these results link the function of Cln5 to folic acid-mediated chemotaxis.

### Autophagic Flux Is Increased in *cln5*-Deficient Cells During Growth

**FIGURE 4:**
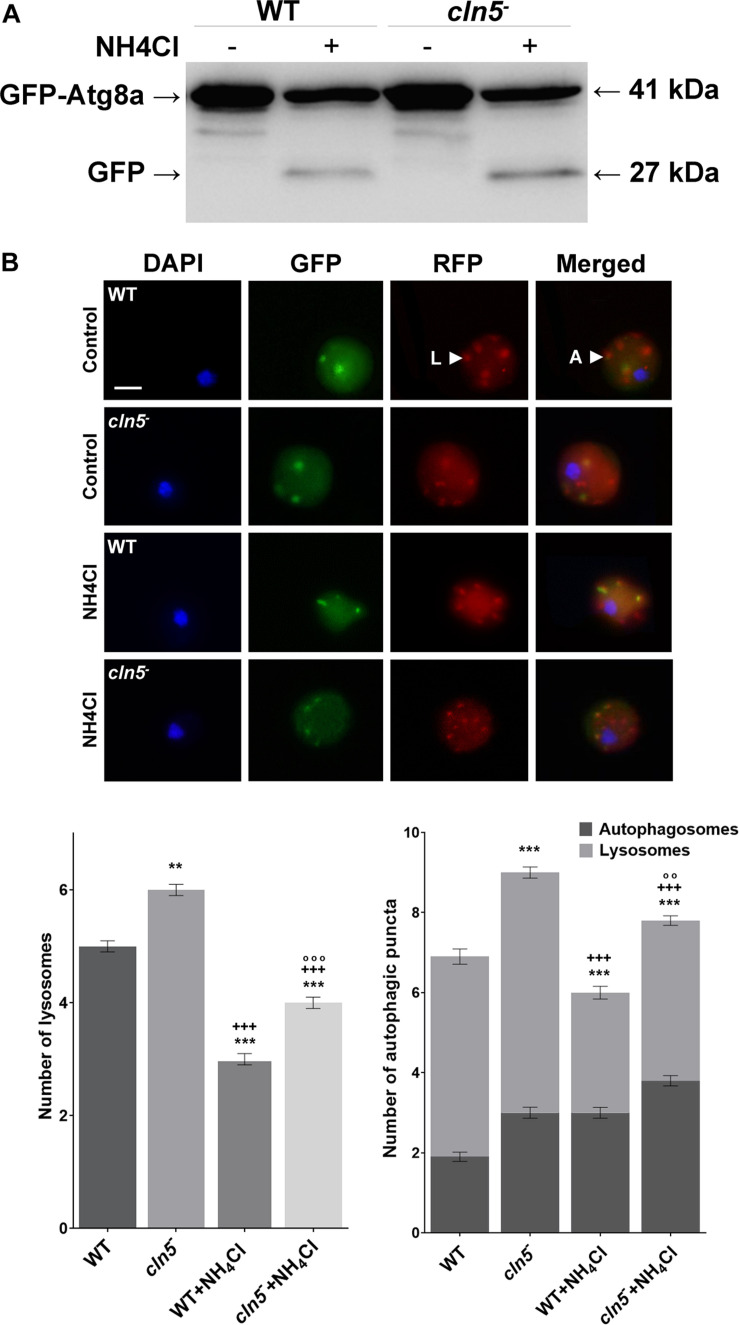
GFP-Atg8a and RFP-GFP-Atg8a autophagic flux assays. **(A)** WT and *cln5*^−^ cells expressing GFP-Atg8a were submerged in HL5 ± ammonium chloride (150 mM) for 4 h. A pulse of ammonium chloride (150 mM) was added halfway through the incubation (after 2 h). Whole cell lysates (30 μg) were separated by SDS-PAGE and analyzed by western blotting using anti-GFP. Molecular weight markers (in kDa) are shown to the right of the blot. Western blot is representative of three independent experiments. **(B)** WT and *cln5*^−^ cells were submerged in low fluorescence HL5 ± ammonium chloride (150 mM) for 4 h, with a pulse of ammonium chloride (150 mM) added halfway through the incubation (after 2 h). Cells were fixed and stained with DAPI to reveal nuclei (blue). Scale bar = 2.5 μm. The average number of autophagosomes and lysosomes was quantified. Data presented as mean number of lysosomes ± SEM (*n* = 3, at least 60 in total were analyzed) and mean total number of autophagic puncta ± SEM (*n* = 3, at least 60 in total were analyzed). Statistical significance was assessed using the non-parametric Kruskal–Wallis test followed by Dunn’s multiple comparisons test. **p < 0.01, ***p < 0.001 vs. WT/^+++^*p* < 0.001 vs. *cln5*^− /^°°*p* < 0.01, °°°*p* < 0.001 vs. WT + ammonium chloride.

Since the previous experiments suggested that Cln5 may play a role in regulating autophagy during growth, we performed two autophagic flux assays; (1) a GFP-Atg8a assay, which provides insight into the number of autophagosomes per cell and (2) an RFP-GFP-Atg8a assay, which measures the number of autophagic puncta per cell and the efficiency of autophagic cargo turnover (Mizushima et al., 2010; Calvo-Garrido et al., 2011; Domínguez-Martín et al., 2017). GFP-Atg8a associates with the inner autophagosomal membrane. When the autophagosome fuses with the lysosome, GFP-Atg8a is cleaved and degraded by lysosomal enzymes. In many organisms, GFP is resistant to autophagic degradation. However, in *Dictyostelium*, GFP is rapidly degraded since *Dictyostelium* cells contain extremely acidic lysosomes (Marchetti et al., 2009; Calvo-Garrido et al., 2011; Domínguez-Martín et al., 2017). Treatment of cells with a non-saturating concentration of ammonium chloride elevates the pH of the lysosome, thereby inhibiting autophagy. This causes free, cleaved GFP to accumulate, which can be visualized by western blotting (Calvo-Garrido et al., 2011; Domínguez-Martín et al., 2017). When WT and *cln5*^–^ cells expressing GFP-Atg8a were incubated in HL5 containing ammonium chloride, *cln5*^–^ cells consistently had higher amounts of free, cleaved GFP suggesting that *cln5*^–^ cells may have had more autophagosomes than WT cells (Figure 4A). This observation could indicate one of two things, that (1) autophagy is induced, or that (2) autophagy is blocked, meaning that autophagosome-lysosome fusion is impaired (Mizushima et al., 2010; Domínguez-Martín et al., 2017). Therefore, to further investigate the autophagic defect in *cln5*^–^ cells, an RFP-GFP-Atg8a autophagic flux assay was performed using WT and *cln5*^–^ cells expressing RFP-GFP-Atg8a. RFP-GFP-Atg8a is incorporated on the inner autophagosomal membrane. When autophagosomes fuse with lysosomes, GFP fluorescence is quenched due to the acidic environment of the lysosome. Thus, lysosomes appear red and autophagosomes appear yellow (overlap of RFP and GFP fluorescence) (Calvo-Garrido et al., 2011; Domínguez-Martín et al., 2017). In this assay, it was observed that *cln5*^–^ cells had more autophagic puncta than WT cells (autophagosomes and lysosomes) (Figure 4B). More specifically, *cln5*^–^ cells had approximately 3 autophagosomes and 6 lysosomes per cell, compared to approximately 2 autophagosomes and 5 lysosomes in WT cells. As in the GFP-Atg8a assay, the increased number of autophagic puncta in *cln5*^–^ cells could mean autophagy is induced or there is a block between the fusion of the autophagosome and lysosome. To further investigate this, WT and *cln5*^–^ cells were treated with ammonium chloride which increases lysosomal pH, thereby suppressing autophagy. This treatment should decrease the number of red puncta (lysosomes) in cells. If the number of red puncta does not decrease after ammonium chloride treatment, then the cells have a block in autophagy, meaning a block in the fusion between the autophagosome and lysosome. In this study, we observed that ammonium chloride reduced the number of red puncta in *cln5*^–^ cells compared to WT cells (Figure 4B). Together, the two autophagic flux assays provide evidence that the basal level of autophagy in *cln5*^–^ cells is higher than in WT cells.

### *cln5*-Deficiency Increases the Amounts of Ubiquitinated Proteins During Growth

**FIGURE 5:**
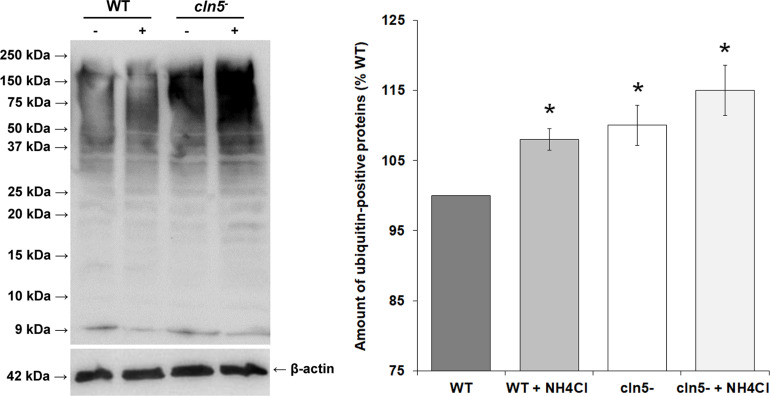
Effect of *cln5*-deficiency on the amounts of ubiquitin-positive proteins. WT and *cln5*^−^ cells were submerged in HL5 ± ammonium chloride (100 mM) for 2 h. Whole cell lysates (25 μg) were separated by SDS-PAGE and analyzed by western blotting with anti-ubiquitin and anti-beta-actin (loading control). Molecular weight markers (in kDa) are shown to the left of the blot. The amounts of ubiquitin-positive proteins were quantified, normalized to beta-actin, and plotted. Raw values were then expressed as a percentage of the amounts of ubiquitin-positive proteins in WT cells. Data presented as the mean amounts of ubiquitin-positive proteins (% WT) ± SEM (*n* = 3). Statistical significance was assessed using the one-sample *t*-test. *p-value < 0.05 vs. WT.

In *Dictyostelium*, ubiquitination regulates several processes and co-localizes with the autophagosomal markers Atg8a and Atg8b (Welchman et al., 2005; Pankiv et al., 2007; Kirkin et al., 2009; Matthias et al., 2016). Since autophagy was dysregulated in *cln5*^–^ cells, we used western blotting to assess the effect of *cln5*-deficiency on the amounts of ubiquitinated proteins in growth-phase cells. *cln5*-deficiency increased the amounts of ubiquitin-positive proteins by 10 ± 3% during growth (Figure 5). When cells were incubated in HL5 containing ammonium chloride for 2 h, the amounts of ubiquitin-positive proteins in both WT and *cln5*^–^ cells increased, which was expected since ammonium chloride inhibits autophagy (Figure 5). Under these conditions, the amounts of ubiquitin-positive proteins were again increased in *cln5*^–^ cells relative to WT cells (Figure 5). These findings support the increased level of basal autophagy in *cln5*^–^ cells and suggest that proteins are being tagged in *cln5*^–^ cells but they are not being broken down effectively.

### Cln5 Plays a Role in Aggregation but Has No Effect on Mound Size

**FIGURE 6:**
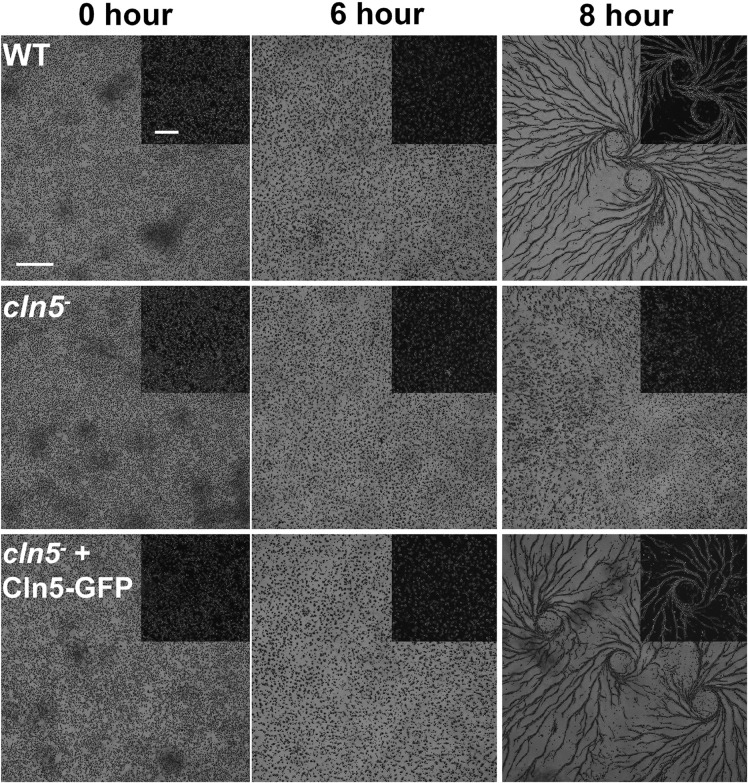
Effect of *cln5*-deficiency on aggregation. WT, *cln5*^–^, and *cln5*^–^ + Cln5-GFP cells were submerged in KK2 buffer and imaged at the indicated times. Scale bar = 500 μm. Insert scale bar = 250 μm.

**FIGURE 7:**
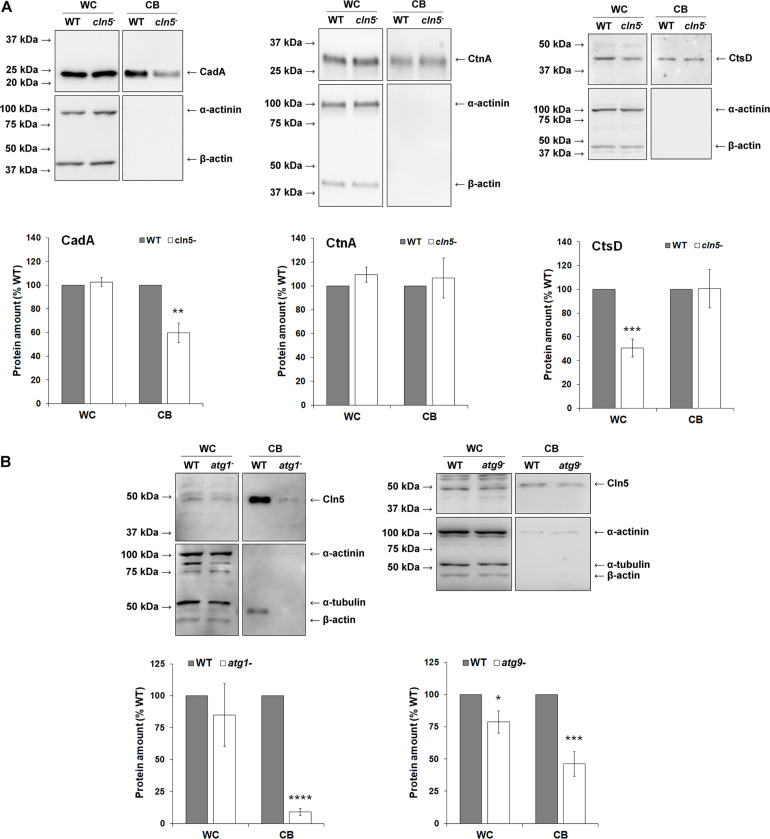
Examining the role and secretion of Cln5 during aggregation. **(A)** Effect of *cln5*-deficiency on the intracellular and extracellular amounts of CadA, CtnA, and CtsD during starvation. WT and *cln5*^−^ cells were starved for 4 h in KK2 buffer after which time the cells were lysed. Whole cell (WC) lysates (25 μg) and conditioned buffer (CB, 0.25 μg) were separated by SDS-PAGE and analyzed by western blotting with anti-CadA, anti-CtnA, anti-CtsD, anti-alpha-actinin (loading control), and anti-beta-actin (loading control). Molecular weight markers (in kDa) are shown to the left of each blot. Protein bands were quantified and standardized to the levels of alpha-actinin and beta-actin. Raw values were then expressed as a percentage of the protein amounts in WT WC lysates and CB. Data presented as mean amount of protein (% WT) ± SEM (*n* ≥ 7). Statistical significance was determined using the one sample *t*-test. **p < 0.01 and ***p < 0.001 vs. WT. **(B)** Assessing the role of Atg proteins in Cln5 secretion. WT, *atg1*^−^, and *atg9*^−^ cells were starved for 4 h in KK2 buffer after which time the cells were lysed. WC lysates (20 μg) and CB (0.2 μg) were separated by SDS-PAGE and analyzed by western blotting with anti-Cln5, anti-alpha-actinin (loading control), anti-alpha-tubulin (loading control), and anti-beta-actin (loading control). Molecular weight markers (in kDa) are shown to the left of each blot. Protein bands were quantified and standardized to the levels of alpha-actinin, alpha-tubulin, and beta-actin. Raw values were then expressed as a percentage of the amount of Cln5 in WT WC lysates and CB. Data presented as mean amount of protein (% WT) ± SEM (*n* ≥ 4). Statistical significance was determined using the one sample *t*-test. ***p < 0.001 and ****p < 0.0001 vs. WT.

As nutrients are not readily available during multicellular development, *Dictyostelium* cells use autophagy to meet the energy demands required for the transition to multicellularity (Mesquita et al., 2017). Previous work showed that *cln5*^–^ cells display reduced adhesion and cAMP-mediated chemotaxis during the early stages of development (Huber and Mathavarajah, 2018b). In human cells, defects in adhesion and chemotaxis have been associated with aberrant autophagy (Coly et al., 2016; Ligon et al., 2020). Since Cln5 was shown to regulate autophagy during growth, and multicellular development relies on autophagic mechanisms, the effect of *cln5*-deficiency during multicellular development was assessed (Otto et al., 2003, 2004). WT, *cln5*^–^, and *cln5*^–^ + Cln5-GFP cells were deposited into Petri dishes and then starved in KK2 buffer. Under these conditions, loss of *cln5* delayed aggregation by ∼2 h (Figure 6). We then explored the effect of *cln5*-deficiency on the intracellular and extracellular amounts of two proteins linked to cell adhesion and aggregation, CadA and CtnA (Brar and Siu, 1993; Brock and Gomer, 1999). Loss of *cln5* decreased the extracellular amount of CadA after 4 h of starvation but had no effect on the amount of extracellular CtnA or the intracellular amounts of either protein (Figure 7A). Since autophagy relies on the activity of lysosomal enzymes, we also examined the effect of *cln5*-deficiency on the intracellular and extracellular amounts of the lysosomal aspartyl protease, cathepsin D (CtsD) (Journet et al., 1999). In humans, mutations in *CTSD* cause a subtype of NCL called CLN10 disease (Tyynelä et al., 2000). Loss of *cln5* significantly decreased the intracellular amount of CtsD but had no effect on the extracellular amount of the protein (Figure 7A). Combined, these data suggest that Cln5 regulates the trafficking of CadA and the intracellular amount of CtsD during the early stages of multicellular development. Previous work showed that pharmacological inhibition of autophagy with ammonium chloride or chloroquine reduces the extracellular amount of Cln5 during starvation (Huber and Mathavarajah, 2018b). To further examine the role of Cln5 in autophagy, we assessed the secretion of Cln5 in cells lacking either *atg1* or *atg9*. Atg1 is a protein kinase that co-localizes with Atg8 and is required for macroautophagy and multicellular development (Otto et al., 2004; Tekinay et al., 2006). Atg9 is a transmembrane protein that localizes to small vesicles and is predicted to play a role in membrane trafficking to autophagosomes (Calvo-Garrido et al., 2010; Tung et al., 2010). Loss of *atg9* disrupts growth, phagocytosis, and morphogenesis during development (Tung et al., 2010; Yamada and Schaap, 2019). Consistent with our previous findings, we observed reduced extracellular amounts of Cln5 in *atg1*^–^ and *atg9*^–^ cells (Figure 7B). To complete our analysis of the effect of *cln5*-deficiency on the early stages of development, we observed no significant effect of *cln5* loss on mound number or size (Supplementary Figure 2). Together, these results show that *cln5*-deficiency delays aggregation by affecting the trafficking of CadA. The role of Cln5 in autophagy is further supported by the impact of *cln5*-deficiency on the intracellular amount of CtsD and the reduced secretion of Cln5 in *atg1*^–^ and *atg9*^–^ cells.

### Cln5 and NagA May Participate in the Same Biological Pathway to Regulate Autophagy

**FIGURE 8:**
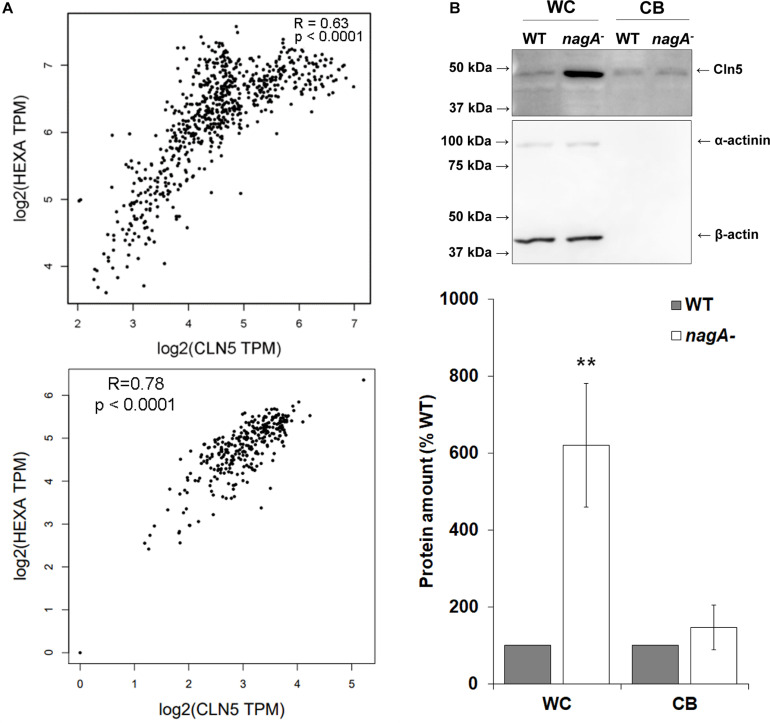
Examining the link between CLN5/Cln5 and HEXA/NagA. **(A)** Co-expression of *CLN5* and *HEXA* in human tissues. RNA sequencing data across 53 non-diseased tissue sites (8,587 samples) were collected from the GTEx Project, which is available on the GEPIA2 database (http://gepia.cancer-pku.cn/index.html). The correlation analysis tool on the GEPIA2 database was used to analyze the expression of *CLN5* and *HEXA* in these samples (top). The correlation analysis tool was also used to analyze the expression of *CLN5* and *HEXA* in a subset of samples from brain tissue (cerebellum, cortex, and hippocampus) (bottom). Pearson correlation statistics were calculated for each plot and the statistical significance for the correlations were determined. Transcripts per million (TPM). **(B)** Effect of *nagA*-deficiency on the intracellular and extracellular amounts of Cln5. Cells were starved for 4 h in KK2 buffer after which time the cells were lysed. Whole cell (WC) lysates (20 μg) and samples of conditioned buffer (CB, 0.2 μg) were separated by SDS-PAGE and analyzed by western blotting with anti-Cln5, anti-alpha-actinin (loading control), and anti-beta-actin (loading control). Molecular weight markers (in kDa) are shown to the left of the blot. Protein bands were quantified and standardized to the levels of alpha-actinin and beta-actin. Raw values were then expressed as a percentage of the amount of Cln5 in WT WC lysates and CB. Data presented as mean amount of protein (% WT) ± SEM (*n* = 7). Statistical significance was determined using the one sample *t*-test. **p-value < 0.01 vs. WT.

To further explore the role of Cln5 in autophagy during the early stages of development, we referenced our previous work that identified a putative N-acetylglucosamine thiazoline (NGT)-binding site in human CLN5 (Huber and Mathavarajah, 2018a). In humans, a well-established NGT-binding protein is the lysosomal enzyme hexosaminidase A (HEXA). HEXA activity is linked to autophagy and mutations in *HEXA* cause the lysosomal storage disorder Tay-Sachs disease (Okada and O’Brien, 1969; Urbanelli et al., 2014; Matsushita et al., 2019). HEXA, CLN5, and *Dictyostelium* Cln5 all have glycoside hydrolase activity (Okada and O’Brien, 1969; Huber and Mathavarajah, 2018a). Based on these findings, we were interested in further exploring the relationship between CLN5 and HEXA. Using the GEPIA database^1^, we found that the expression profiles of *CLN5* and *HEXA* are highly correlated in human tissues (8,587 samples from a collective overview of 53 non-diseased tissue sites) (Tang et al., 2019; Figure 8A). Expression is also highly correlated in brain tissues where *CLN5* is highly expressed and most affected by CLN5 disease (cortex, cerebellum, and hippocampus) (Heinonen et al., 2000; Holmberg et al., 2004; Figure 8A). These observations, coupled with the glycoside hydrolase activity of CLN5 and HEXA, suggested that the two proteins may participate in the same biological pathway and that there may be a potential overlap in how autophagy is disrupted in Tay-Sachs disease and the NCLs. To examine this possibility in *Dictyostelium*, we assessed the amounts of intracellular and extracellular Cln5 in cells lacking *N*-acetylglucosaminidase (*nagA*), the *Dictyostelium* homolog of human HEXA (Dimond et al., 1973). Loss of *nagA* increased the intracellular amount of Cln5 by over 500% in cells starved for 4 h but had no effect on the amount of extracellular Cln5 suggesting that *nagA*-deficient cells upregulate *cln5*, potentially as a compensatory mechanism to increase glycoside hydrolase activity (Figure 8B). In total, these results suggest that CLN5/Cln5 and HEXA/NagA may participate in the same pathway to regulate autophagy, which is consistent with their shared function as glycoside hydrolases.

### Loss of *cln5* Accelerates Tipped Mound, Slug, and Fruiting Body Formation

**FIGURE 9:**
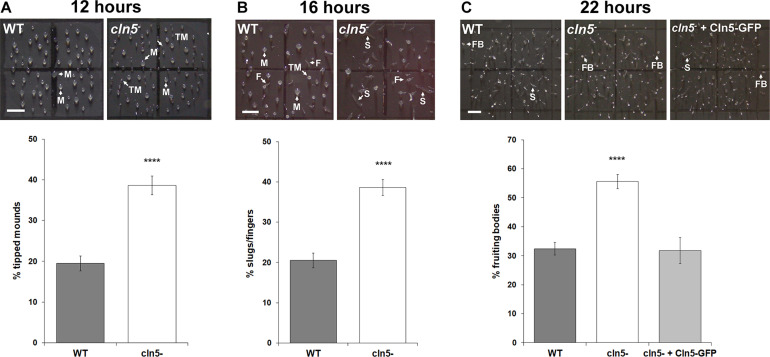
Effect of *cln5*-deficiency on the mid-to-late stages of development. **(A)** Effect of *cln5*-deficiency on tipped mound formation after 12 h of development. Data presented as mean percentage of tipped mounds ± SEM (*n* > 84). **(B)** Effect of *cln5*-deficiency on finger and slug formation after 16 h of development. Data presented as mean percentage of fingers/slugs ± SEM (*n* > 88). **(C)** Effect of *cln5*-deficiency on fruiting body formation after 22 h of development. Data presented as mean percentage of fruiting bodies ± SEM (*n* > 32). Arrows (white) indicate mounds (M), tipped mounds (TM), slugs (S), fingers (F), and fruiting bodies (FB). Scale bar = 1 mm. Statistical significance for panels **(A,B)** was determined using the Student’s *t*-test. Statistical significance for panel **(C)** was determined using one-way ANOVA followed by Tukey’s multiple comparisons test. ****p-value < 0.0001 vs. WT.

CLN5 has been linked to brain development in mice and humans (Heinonen et al., 2000; Holmberg et al., 2004; Fabritius et al., 2014; Singh et al., 2019). In addition, recent work in *Cln5*-deficient mice speculated that CLN5 function is linked to neurogenesis (Savchenko et al., 2017). We were therefore interested in using *Dictyostelium* to further study the role of CLN5 in developmental processes. Following aggregation, *cln5*^–^ cells formed tipped mounds prior to WT cells (39 ± 2% of *cln5*^–^ mounds were tipped after 12 h of development compared to 19 ± 2% of WT mounds) (Figure 9A). We then observed that development of *cln5*^–^ cells into fingers, slugs, and fruiting bodies was also precocious. More specifically, after 16 h of development, 38 ± 2% of *cln5*^–^ multicellular structures were fingers and slugs compared to 21 ± 2% of WT multicellular structures (Figure 9B). After 22 h of development, 55 ± 2% of *cln5*^–^ multicellular structures developed into fruiting bodies compared to 32 ± 2% of WT multicellular structures (Figure 9C). Introducing Cln5-GFP into *cln5*^–^ cells suppressed the precocious development of *cln5*^–^ cells into fruiting bodies providing evidence that the accelerated development of *cln5*^–^ cells was due to the absence of the Cln5 protein in mutant cells (Figure 9C). Intriguingly, these findings are consistent with observations in *Dictyostelium* knockout models of CLN2 and CLN3 disease suggesting that a similar pathway may be perturbed in the three knockout models (*tpp1A*^–^, *cln3*^–^, and *cln5*^–^) (Huber et al., 2014; Phillips and Gomer, 2015). Together, these results show that *cln5*-deficiency affects developmental timing in *Dictyostelium*.

### Loss of *cln5* Affects Spore Morphology, Germination, and Viability

Autophagy is known to regulate terminal differentiation in *Dictyostelium* (Kin et al., 2018; Yamada and Schaap, 2019, 2020). This knowledge, combined with the precocious development of *cln5*^–^ cells, led us to investigate the effects of *cln5*-deficiency on spore morphology, germination, and viability. Loss of *cln5* had no effect on spore area (Table 1). However, *cln5*^–^ spores were more rounded compared to WT spores (Table 1). We also assessed the effect of *cln5*-deficiency on the timing of spore germination and observed that the time required for *cln5*^–^ spores to germinate was increased by ∼30 min compared to WT spores (Table 1). Finally, we used a plaque assay to assess spore viability. When WT spores were plated with bacteria on SM/2 agar, 70 ± 4% of the spores formed plaques, compared to only 49 ± 2% of *cln5*^–^ spores (Table 1). Expression of Cln5-GFP in *cln5*^–^ cells rescued the defects in spore roundedness, germination, and viability suggesting that Cln5 regulates these processes in *Dictyostelium* possibly via its role in autophagy.

**TABLE 1:** Effect of *cln5*-deficiency on spore area, morphology, germination, and viability.

	WT	*cln5*^–^	*cln5*^–^ + Cln5-GFP
Normalized spore area	0.048 ± 0.002	0.048 ± 0.002	0.047 ± 0.003
Spore roundedness	0.63 ± 0.010	0.70 ± 0.003*	0.65 ± 0.005
Timing of spore germination (min)	358 ± 3	390 ± 6****	351 ± 3
Spore viability (% initial spores plated)	70 ± 4	49 ± 1****	69 ± 8

All data presented as mean ± SEM (n = 3–4). At least 300 spores were analyzed for each biological replicate. Details on the methods and statistical analyses performed are found in section “Spore Morphology, Germination, and Viability.” *p-value < 0.05 and ****p-value < 0.0001 vs. WT.

### Aberrant Autophagy Underlies the Development of *cln5*^–^ Cells

**FIGURE 10:**
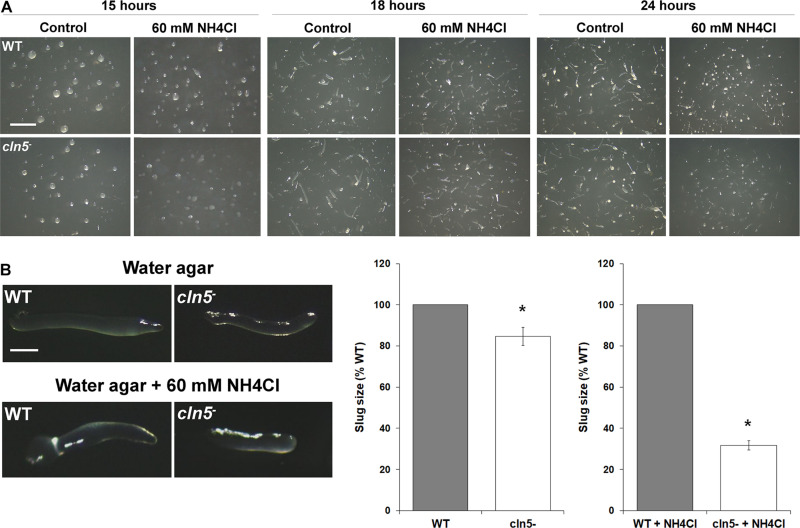
Effect of *cln5*-deficiency on multicellular development when autophagy is inhibited. **(A)** WT and *cln5*^−^ cells were deposited on 0.5% agar/distilled water ± ammonium chloride (60 mM). Images were taken at the mound/tipped mound (15 h), finger/slug (18 h), and fruiting body (24 h) stages of development. Images are representative of three independent experiments. Scale bar = 2.5 mm. **(B)** Effect of *cln5*-deficiency on slug size ± ammonium chloride (60 mM). Scale bar = 50 μm. Raw values were then expressed as a percentage of WT slug size. Data presented as mean slug size (% WT) ± SEM (*n* = 3, at least eight slugs were quantified for each biological replicate). Statistical significance was determined using the one-sample *t*-test. *p-value < 0.05 vs. WT.

*Dictyostelium* autophagy mutants display a variety of abnormal developmental phenotypes (Otto et al., 2004). These defects are more prominent during development on agar as opposed to nitrocellulose filters, and even more pronounced when autophagy is inhibited (Otto et al., 2003, 2004; Kosta et al., 2004). Therefore, we monitored the development of WT and *cln5*^–^ cells on distilled water agar containing ammonium chloride. *cln5*^–^ development was more impaired compared to WT in the presence of ammonium chloride. *cln5*^–^ mounds appeared smaller and flatter than WT mounds (Figure 10A). Slugs formed by *cln5*^–^ cells were smaller and translucent, making them difficult to see with the unaided eye (Figure 10B). Slug size was reduced by 15 ± 4% on distilled water agar and by 68 ± 2% on distilled water agar containing ammonium chloride, demonstrating that the phenotype was exaggerated when autophagy was inhibited (Figure 10B). Lastly, fruiting body stalks appeared shorter and delicate, while sori appeared smaller (Figure 10A). The impaired development of *cln5*^–^ cells in autophagy-inhibiting conditions provides additional evidence linking Cln5 to autophagy during *Dictyostelium* development.

## Discussion

The aim of this study was to better understand the function of Cln5 in *Dictyostelium*. During growth, *cln5*-deficiency inhibited cell proliferation and cytokinesis but had no effect on pinocytosis. Loss of *cln5* also compromised proliferation and viability in nutrient-limiting media and inhibited folic acid-mediated chemotaxis. GFP-Atg8a and RFP-GFP-Atg8a autophagic flux assays revealed that *cln5*-deficiency increased the numbers of autophagic puncta (autophagosomes and lysosomes) within cells, suggesting that loss of *cln5* increases the basal level of autophagy during growth. *cln5*-deficiency also increased the amounts of ubiquitin-positive proteins. During multicellular development, *cln5*-deficiency delayed aggregation. However, following mound formation, the development of *cln5*^–^ cells was precocious. *cln5*^–^ spores displayed aberrant morphology, germination, and viability. The involvement of Cln5 in many of these processes was validated by expressing Cln5-GFP in *cln5*^–^ cells to rescue the gene-deficiency phenotypes.

Loss of *cln5* reduced the rate of cell proliferation in nutrient-rich media and the maximum titer of cultures. In addition, the proliferation and viability of *cln5*^–^ cells was severely compromised in nutrient-limiting media. These observations are consistent with work that examined CLN5 function in mammalian cells. For example, silencing *CLN5* (via RNAi) causes G0/1 arrest in *HeLa* cells, reduces cell number in clear cell renal cell carcinoma, and reduces the viability of HEK293 and primary endothelial cells (Kittler et al., 2007; Gerlinger et al., 2012; Warner et al., 2014; de Rojas-P et al., 2020). However, there may be a cell type-dependent role for CLN5 in regulating cell proliferation since neural progenitor cells derived from a *Cln5*^–/–^ mouse display increased cell proliferation (Savchenko et al., 2017). Therefore, future work is required to clarify the precise role of CLN5 in regulating cell proliferation.

Consistent with the reduced proliferation of *cln5*^–^ cells, we also observed a mild defect in cytokinesis. Interestingly, Cln3 regulates the secretion of Cln5 in *Dictyostelium*, and we observed similar cytokinesis defects in *cln3*^–^ cells (Huber and Mathavarajah, 2018b; Mathavarajah et al., 2018). In addition, our previous work revealed that Cln5 has glycoside hydrolase activity (Huber and Mathavarajah, 2018a). Together, these findings suggest that Cln5 may promote cytokinesis in *Dictyostelium* by cleaving glycosides outside the cell to alter the polysaccharide content in the extracellular space. This is supported by work in *Caenorhabditis elegans* showing that secreted proteins can influence cytokinesis by altering the polysaccharide composition of the extracellular matrix (Vogel et al., 2011; Xu and Vogel, 2011).

In this study, we showed that the reduced proliferation of *cln5*^–^ cells was not due to decreased intake of nutrients via pinocytosis. This finding suggests that while *cln5*^–^ cells can internalize nutrients normally, the ability of cells to process those nutrients may be compromised. This was supported by our findings that loss of *cln5* increases the basal level of autophagy during growth, which may reflect a cellular approach to compensate for the reduced degradation of internal resources in *cln5*^–^ cells. CLN5 has also been linked to autophagy in mammalian systems. Retinal extracts from *Cln5*-deficient mice have increased amounts of LC3-II and other proteins involved in autophagosome formation (Leinonen et al., 2017). CLN5 disease patient fibroblasts and *CLN5*-deficient *HeLa* cells have increased LC3-II and an increased number of basal autophagic puncta, specifically lysosomes, suggesting autophagy is induced in those experimental systems, which is consistent with our findings in *Dictyostelium* (Adams et al., 2019). Finally, work in cellular and murine models of CLN5 disease suggests that CLN5 may play a role in activating mitophagy (Doccini et al., 2020).

Upon activation of the autophagy pathway, polyubiquitinated proteins and inclusion bodies are delivered to the autophagosome for degradation. When autophagy is disrupted, so is the clearance of proteins tagged by ubiquitin (Hara et al., 2006; Komatsu et al., 2006; Nezis et al., 2008; Kocaturk and Gozuacik, 2018). In *Dictyostelium* and other cell models, aberrant autophagic flux is coupled with the accumulation of ubiquitin-positive protein aggregates, which could explain why we observed increased amounts of ubiquitinated proteins in *cln5*^–^ cells (Calvo-Garrido et al., 2010; Tung et al., 2010; Arhzaouy et al., 2012; Muñoz-Braceras et al., 2015; Xiong et al., 2015; Meßling et al., 2017). Since we observed an increased level of basal autophagy in *cln5*^–^ cells, it is also possible that the increased amounts of ubiquitinated proteins could reflect an attempt by *cln5*^–^ cells to generate more nutrients. However, future work will be required to determine why ubiquitin-positive proteins are increased in *cln5*^–^ cells.

In addition to aberrant proliferation and cytokinesis, *cln5*^–^ cells also displayed reduced folic acid-mediated chemotaxis, which allows amoebae to detect and internalize their food source during the growth phase of the life cycle. In *Dictyostelium*, the G protein-coupled folic acid receptor mediates both chemotaxis toward folic acid and the phagocytosis of bacteria (Pan et al., 2016). In HEK-293 and U87 glioblastoma cells, chemotactic G protein-coupled receptors have been shown to control cell migration by repressing autophagosome biogenesis (Coly et al., 2016). Therefore, these findings suggest that the reduced chemotaxis of *cln5*^–^ cells toward folic acid may have been due to alterations in the autophagy pathway.

In this study, we revealed that loss of *cln5* delays aggregation, which could be explained by the previously reported effects of *cln5*-deficiency on cAMP-mediated chemotaxis and adhesion (Huber and Mathavarajah, 2018b). Interestingly, these phenotypes have also been reported in *cln3*^–^ cells (Huber et al., 2017). Consistent with these findings, we revealed that loss of *cln5* altered the extracellular amount of the calcium-dependent cell adhesion protein CadA. We also observed no effect of *cln5*-deficiency on the intracellular and extracellular levels of CtnA. Since CtnA regulates aggregate size, it was not surprising that loss of *cln5* had no effect on aggregate number or size (Brock and Gomer, 1999). Like growth, the delayed aggregation of *cln5*^–^ cells could also be explained by aberrant autophagy. This is supported by our observation that loss of *cln5* reduced the intracellular amount of the lysosomal enzyme CtsD. In addition, we revealed reduced secretion of Cln5 in *atg1*^–^ and *atg9*^–^ cells suggesting that the trafficking of Cln5 is dependent on the activities of these two autophagy proteins. Atg1 is a protein kinase that is required for macroautophagy and co-localizes with Atg8a, which was used as a marker of autophagic flux in this study (Otto et al., 2004; Tekinay et al., 2006). Atg9 localizes to small vesicles and is predicted to play a role in membrane trafficking to autophagosomes (Calvo-Garrido et al., 2010; Tung et al., 2010). Importantly, these findings adhere to previous work that reported reduced secretion of Cln5 when WT cells were treated with ammonium chloride or chloroquine, two inhibitors of autophagy (Huber and Mathavarajah, 2018b).

Loss of *cln5* accelerated the mid-to-late stages of *Dictyostelium* development, which has also been reported for *tpp1A*^–^ and *cln3*^–^ cells (Huber et al., 2014; Phillips and Gomer, 2015). Precocious development is a common phenotype of *Dictyostelium* autophagy mutants and can be explained by the inability of cells to either sense nutrients in their environment or process nutrients that have been internalized (Mesquita et al., 2017). This prematurely places cells in a starved state and causes them to progress through the developmental program faster. *cln5*^–^ development was also impaired when cells were placed on distilled water agar containing ammonium chloride, an inhibitor of autophagy. Since ammonium chloride treatment suppressed the precocious development of *cln5*^–^ cells, this finding aligns with the increased basal level of autophagy observed in *cln5*^–^ cells. In addition, *cln5*-deficient slugs were smaller than WT slugs when developed on distilled water agar. This phenotype was exaggerated on distilled water agar containing ammonium chloride further supporting the role of Cln5 in autophagy. Previous work showed that *tpp1A*^–^ and *cln3*^–^ cells are also sensitive to ammonium chloride during development (Phillips and Gomer, 2015; Mathavarajah et al., 2018). In addition, *tpp1A*^–^ development was impaired by chloroquine, another autophagy-inhibiting chemical (Phillips and Gomer, 2015). These findings support mounting evidence that aberrant autophagy may be a common phenotype underlying all forms of NCL (Huber, 2020). It is important to note that while ammonium chloride is routinely used to inhibit autophagy in *Dictyostelium*, it has also been shown to affect cAMP signaling during *Dictyostelium* development (Williams et al., 1984). Therefore, at this time, we cannot discount the possibility that the observed effects of ammonium chloride on development may have been due to altered cAMP signaling. Nonetheless, our observations show clear differences between WT and *cln5*^–^ development in the presence of ammonium chloride. Finally, while there are significant differences between *Dictyostelium* and mammalian development, the impact of *cln5*-deficiency on multicellular development in *Dictyostelium* mirrors observations in mammalian systems that have linked CLN5 to brain development and neurogenesis (Heinonen et al., 2000; Holmberg et al., 2004; Fabritius et al., 2014; Savchenko et al., 2017; Singh et al., 2019).

Autophagy regulates terminal differentiation in *Dictyostelium* (Kin et al., 2018; Yamada and Schaap, 2019, 2020). Not surprisingly, we observed that loss of *cln5* impacted spore shape, viability, and the timing of germination. Previous work showed that acyl coenzyme A (CoA) binding protein (AcbA), an essential protein in spore formation, is trafficked via autophagosomes and secreted unconventionally, after which it is processed into spore differentiation factor 2 (SDF-2) (Duran et al., 2010). The defects in the shape of *cln5*^–^ spores may possibly be explained by defects in the autophagy-mediated trafficking of key proteins required for spore formation, thus contributing to altered spore shape and reduced viability. In addition, ultrastructural images taken during spore germination suggest that autophagic vacuoles play an important role in *Dictyostelium* spore germination (Cotter et al., 1969). Therefore, the autophagy defects in *cln5*^–^ cells may have contributed to the reduced germination of spores.

Previous work showed that *Dictyostelium* Cln5 and human CLN5 have glycoside hydrolase activity (Huber and Mathavarajah, 2018a). A well-characterized glycoside hydrolase in humans is HEXA, which localizes to the lysosome and is associated with Tay-Sachs disease (Okada and O’Brien, 1969). Not surprisingly, HEXA has also been linked to autophagy (Urbanelli et al., 2014; Matsushita et al., 2019). In this study, we showed that the expression profiles of *HEXA* and *CLN5* are highly correlated in human tissues, particularly in brain tissue most affected by CLN5 disease. We then showed that loss of *nagA*, the *Dictyostelium* homolog of *HEXA*, dramatically increases the intracellular amount of Cln5 during starvation. Since the extracellular amount of Cln5 was unaffected by the loss of nagA these findings suggest that *nagA*^–^ cells increase the expression of *cln5* to compensate for the reduced glycoside hydrolase activity in *nagA*-deficient cells. These observations not only provide additional evidence supporting the glycoside hydrolase activity of Cln5, but they also suggest that Cln5 and NagA may participate in the same biological pathway. This is further supported by previous work that identified NagA as a Cln5-interactor (Huber and Mathavarajah, 2018a). In addition, like *cln5*-deficiency, loss of *nagA*, also reduces slug size in *Dictyostelium* (Dimond et al., 1973). Together, these important findings provide additional insight into the molecular function of CLN5 and the pathological mechanisms that may underlie CLN5 disease.

The online bioinformatics resource for the *Dictyostelium* research community, dictyBase, maintains a database listing the phenotypes of knockout mutants that have been generated by the community. For the purposes of this study, we identified those mutants that were characterized as displaying aberrant autophagy, increased autophagy, or an increased number of autophagosomes (Supplementary Table 1). We then compared the additional phenotypes revealed in those mutants to the growth and developmental phenotypes of *cln5*^–^ cells to gain insight into the pathways that may mediate the function of Cln5 in *Dictyostelium*. Not surprisingly, this analysis revealed many autophagy genes (e.g., *atg1*^–^, *atg5*^–^, *atg6*^–^, *atg7*^–^, *atg8*^–^, *atg9*^–^, and *atg13*^–^), providing additional evidence linking Cln5 to the autophagy pathway. Common phenotypes displayed by autophagy mutants include decreased or abolished growth and aberrant fruiting body development, which were both observed in *cln5*^–^ cells. Of the mutants identified, *ino1*^–^ cells share several common phenotypes with *cln5*^–^ cells, including an increased number of autophagosomes, abolished growth, aberrant cytokinesis, decreased cell-substrate adhesion, and aberrant fruiting body morphology (Frej et al., 2016). *ino1* encodes inositol-3-phosphate synthase, which is involved in inositol phosphate metabolism and is linked to many human diseases including diabetes, bipolar disorder, and Alzheimer’s disease (McLaurin et al., 1998; Silverstone et al., 2005; Fischbach et al., 2006; Scioscia et al., 2007). Altered inositol levels have also been reported in CLN2 disease patients and a CLN3 disease mouse model (Seitz et al., 1998; Pears et al., 2005). Combined, these findings suggest that Cln5 may play a role in maintaining inositol homeostasis within cells.

In summary, this study reports several growth and developmental phenotypes in *cln5*^–^ cells that are associated with aberrant autophagy, which supports mounting evidence in other systems linking the NCLs to the autophagy pathway. However, it is important to note that some of our observations of *cln5*^–^ cells, including aberrant cytokinesis, chemotaxis, and aggregation, could be due to defects in cytoskeletal components. Therefore, while the sum of our data suggest that autophagy is compromised in *cln5*^–^ cells, the impact of *cln5*-deficiency on the cytoskeleton should be examined in future studies. We previously showed that *Dictyostelium* Cln5 and human CLN5 have glycoside hydrolase activity (Huber and Mathavarajah, 2018a). Since both proteins are secreted, another possible explanation for *cln5*-deficient phenotypes is that Cln5 generates a signaling molecule required for *Dictyostelium* growth and development. This is consistent with observations that secreted proteins can influence developmental timing (Chubb et al., 2006). However, additional work is required to confirm this hypothesis. Since the molecular function of CLN5 is conserved between humans and *Dictyostelium*, studying the multifaceted role of Cln5 during *Dictyostelium* growth and development could provide valuable new insight into the function of CLN5 in human cells. As the pathological mechanism of CLN5 disease is unknown, this work provides exciting new insight into the cellular mechanisms that may contribute to CLN5 disease, and on a larger scale, all subtypes of NCL.

## Data Availability Statement

The original contributions presented in the study are included in the article/Supplementary Material, further inquiries can be directed to the corresponding author/s.

## Author Contributions

RH: conceptualization, writing–review and editing, funding acquisition, and supervision. MM, SM, WK, SY, and RH: experimentation and data analysis. MM, SM, and RH: writing–original draft. All authors read and approved the final draft of the manuscript.

## Conflict of Interest

The authors declare that the research was conducted in the absence of any commercial or financial relationships that could be construed as a potential conflict of interest.

## Footnotes

1 http://gepia.cancer-pku.cn/index.html

## Abbreviations

cAMP: cyclic adenosine monophosphate
CadA: calcium-dependent cell adhesion molecule A
CLN: ceroid lipofuscinosis neuronal
CtnA: countin
CtsD: cathepsin D
FM: minimal media
HEXA: hexosaminidase A
HL5: nutrient-rich media
NagA: *N*-acetylglucosaminidase A
NCL: neuronal ceroid lipofuscinosis
NGT: N-acetylglucosamine thiazoline
TPP1: tripeptidyl peptidase 1
WT: wild type.
