# Supplementary figures and images for: Aberrant Autophagy Impacts Growth and Multicellular Development in a Dictyostelium Knockout Model of CLN5 Disease
Source: Front Cell Dev Biol. 2021 Jul 5;9:657406. doi: 10.3389/fcell.2021.657406 (PMC8287835; doi:10.3389/fcell.2021.657406)

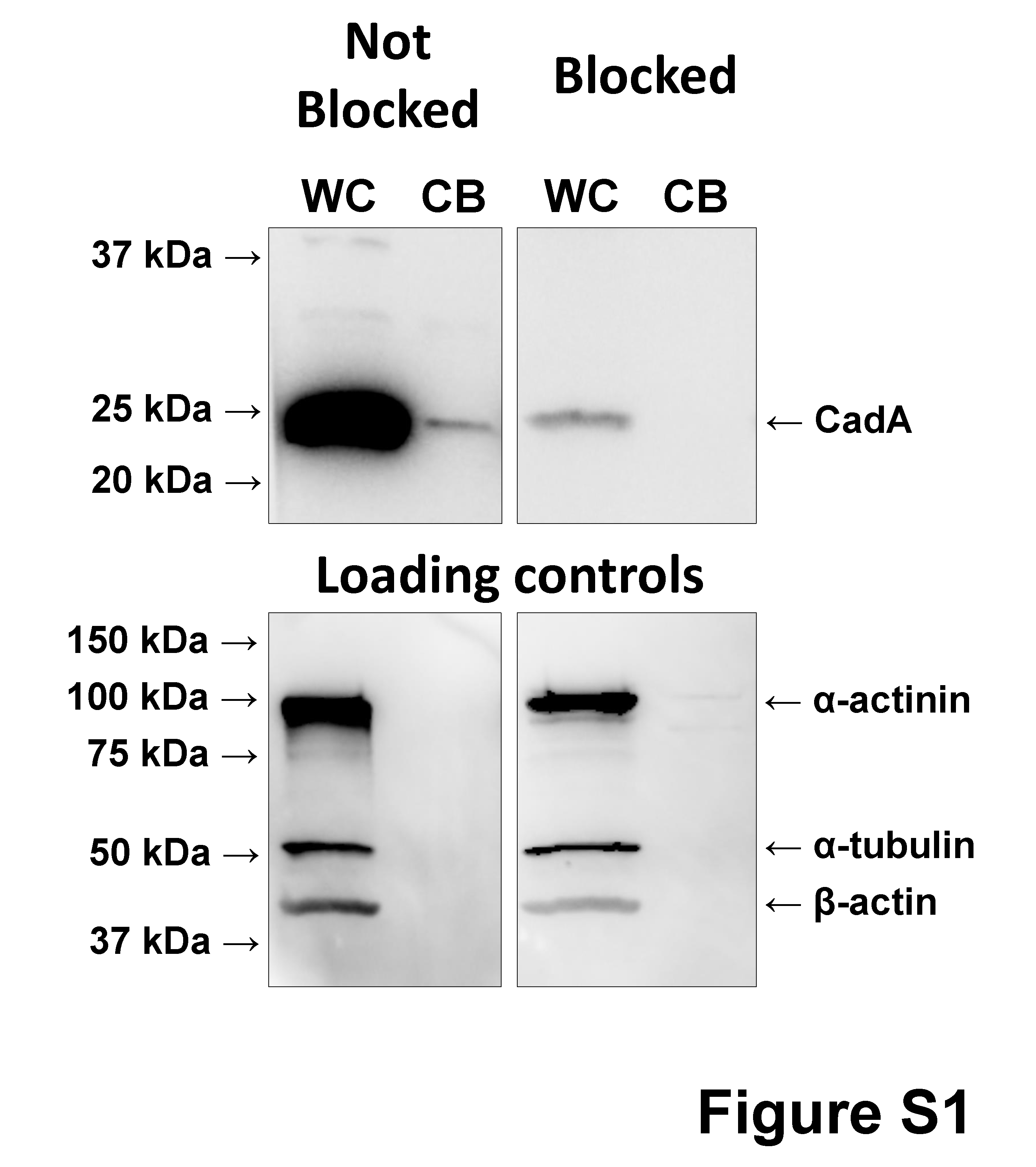

Supplement: Supplementary file 1 [file Image_1.tiff]

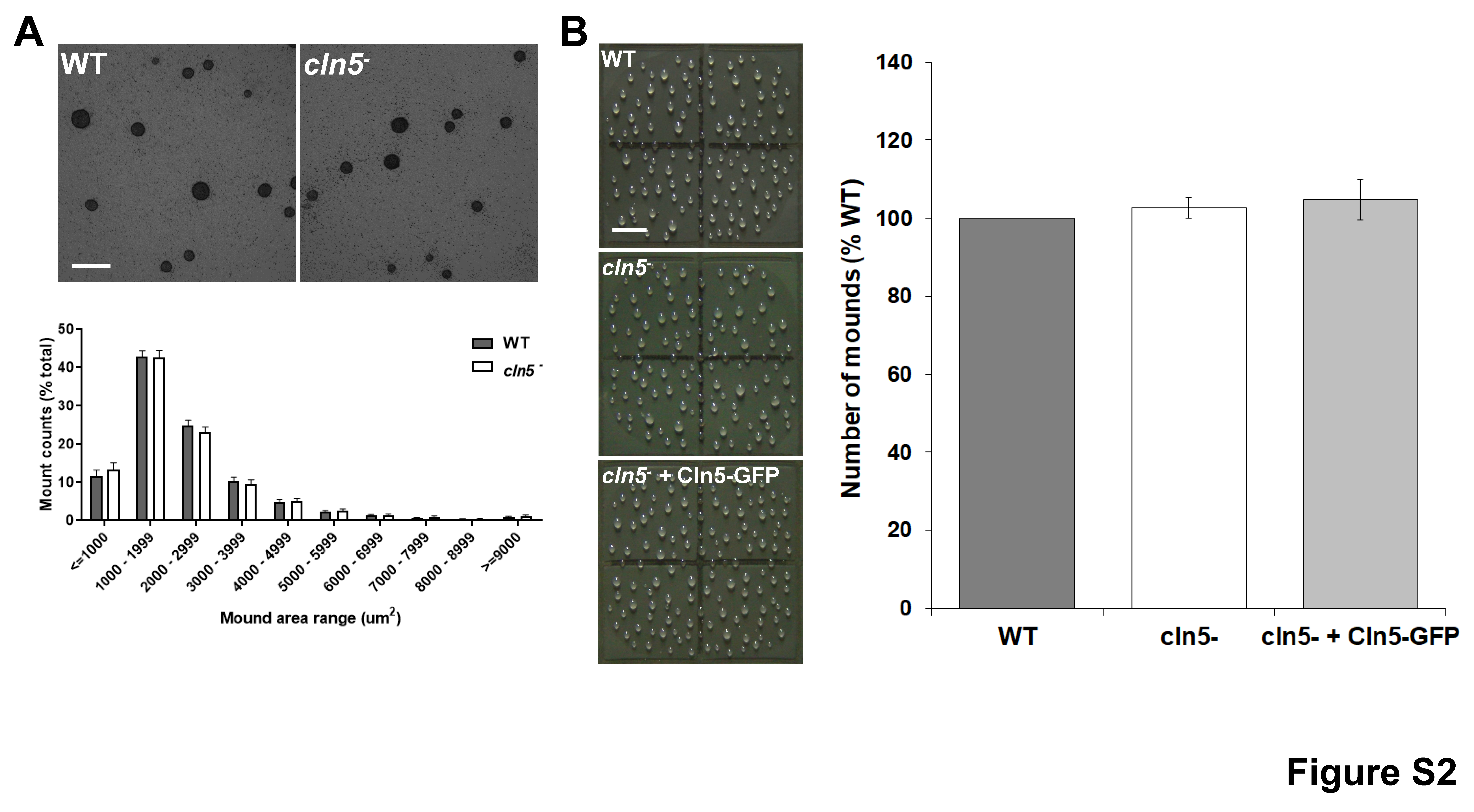

Supplement: Supplementary file 2 [file Image_2.tiff]
