# Supplementary material for: Aberrant Autophagy Impacts Growth and Multicellular Development in a Dictyostelium Knockout Model of CLN5 Disease
Source: Front Cell Dev Biol. 2021 Jul 5;9:657406. doi: 10.3389/fcell.2021.657406 (PMC8287835; doi:10.3389/fcell.2021.657406)
Supplement: Supplementary file 4 [file Data_Sheet_1.docx]

**Supplementary Material**

**Supplemental Figure Captions**

**Fig. S1. Validation of anti-CadA.** WT cells were starved for 4 hours in KK2 buffer. Whole cell lysates (WC, 50 µg) and samples of conditioned buffer (CB, 0.5 µg) were separated by SDS-PAGE and analyzed by western blotting with anti-CadA (not blocked, left) and anti-CadA that was incubated with 500 µg CadA peptide epitope (blocked, right) prior to western blotting. Blots were also probed with the following loading controls: anti-alpha-actinin, anti-alpha-tubulin, and anti-beta-actin. Molecular weight markers (in kDa) are shown to the left of each blot.

**Fig. S2. Effect of *cln5*-deficiency on mound formation. (A)** Effect of *cln5*-deficiency on mound size after 24 hours. Scale bar = 250 µm. Mound areas were placed into bins (µm^2^). Data presented as mean mound counts (% total) ± SEM (n = 28). **(B)** Effect of *cln5*-deficiency on mound formation after 12-15 hours when cells were deposited on nitrocellulose filters soaked in KK2 buffer. Scale bar = 2 mm. Data presented as mean number of mounds (% WT) ± SEM (n ≥ 24).

**Table S1. List of *Dictyostelium* knockout mutants that share phenotypes with *cln5^-^* cells.** Information was obtained from dictyBase and is accurate as of July 28, 2020. Shared phenotypes are indicated in green.
